# Zona otitique, aspects cliniques et thérapeutiques: à propos d’un cas

**DOI:** 10.11604/pamj.2022.41.171.33711

**Published:** 2022-03-03

**Authors:** Ahmed Rouihi, Noureddine Errami, Bouchaib Hemmaoui, Fouad Benariba

**Affiliations:** 1 Service d’Otorhinolaryngologie et de Chirurgie Cervico-Faciale de l’Hôpital Militaire d’Instruction Mohamed V de Rabat, Faculté de Médecine et Pharmacie Rabat, Université Mohamed V Rabat, Rabat, Maroc

**Keywords:** Zona auriculaire, paralysie faciale périphérique, Ramsay-Hunt, cas clinique, Herpes zoster oticus, peripheral facial paralysis, Ramsay-Hunt, case report

## Abstract

Le zona auriculaire, également appelé « herpes zoster oticus » correspond à une infection virale par varicella-zoster virus (VZV) de l'oreille externe, moyenne et/ou interne. L'atteinte de l'oreille associée à une paralysie faciale correspond au syndrome de Ramsay-Hunt dont le diagnostic est clinique. Nous rapportons le cas d´un patient âgé de 25 ans qui a présenté un zona otitique associe à une paralysie faciale périphérique sans autres signes associés. A la lumière de cette observation et d´une revue de la littérature, on discutera les différents aspects cliniques, para-cliniques et évolutifs du zona otitique ainsi que l´attitude thérapeutique.

## Abstract (en)

Ear shingles, also called herpes zoster oticus, are a viral infection of the outer, middle and/or inner ear due to varicella zoster virus (VZV). Patients with ear involvement associated with facial palsy are clinically diagnosed with Ramsay-Hunt´s syndrome. We here report the case of a 25-year-old patient with herpes zoster oticus associated with peripheral facial palsy, without other associated signs. This study and literature review investigate the various clinical, para-clinical and evolutionary features of herpes zoster oticus and the therapeutic approaches.

## Introduction

Décrit par la première fois en 1907 par James Ramsay Hunt, le zona otitique (ou Otitis zona) est une manifestation de récurrence (réactivation) du virus varicelle-zona affectant le ganglion géniculé, secondaire à une diminution de l´immunité à médiation cellulaire. Il est caractérisé par son polymorphisme clinique, son évolution spontanément résolutive, parfois grevée de séquelles ou de complications [1].

## Patient et observation

**Information du patient**: il s´agit d´un patient âgé de 25 ans, diabétique de type I sous insuline qui a présenté une paralysie faciale périphérique droite évoluant depuis deux jours précédée par une otalgie, une hypoacousie et une otorrhée droite.

**Figure 1:**
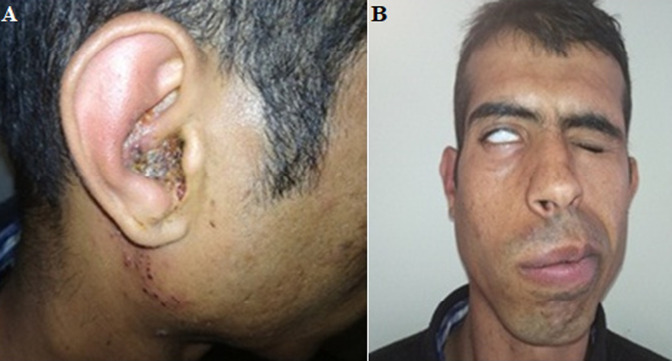
(A) zona de la région de Ramsay Hunt droite; (B) paralysie faciale périphérique droite

**Résultats cliniques**: à l'examen physique, on trouvait une tuméfaction inflammatoire du pavillon, des vésicules au niveau de la conque (Figure 1), l´examen otoscopique trouvait un conduit auditif externe inflammatoire et un tympan congestif sans syndrome vestibulaire, l´examen ophtalmologique n´a pas objectivé de lésions kératite. Le reste de l'examen neurologique et somatique était sans anomalies.

**Démarche diagnostique**: l'audiométrie tonale a révélé une surdité de transmission droite avec un réflexe stapédien aboli. Sur le plan biologique, il avait un syndrome inflammatoire biologique et un diabète déséquilibré, l´examen ophtalmologique na pas objectivé de lésions de kératites d´exposition.

**Intervention thérapeutique et suivi**: le patient a été mis sous Aciclovir intraveineux à la dose de 10 mg/kg toutes les 8 heures pendant 10 jours, avec un relais per os de 7 jours associé à une corticothérapie à la dose de 1 mg/kg/j en cure courte de 10 jours, un antalgique palier 2 et des soins locaux auriculaires et oculaires pour éviter la kératite d´exposition. La rééducation de la paralysie faciale était précoce, le patient a bénéficié de 10 séances de rééducation fonctionnelle pour lutter contre l´hypertonie et les syncinésies avec un suivi psychologique et endocrinien pour équilibration de son diabète. L'évolution à 9 mois était marquée par une récupération totale de la paralysie faciale et une nette amélioration de de la surdité.

## Discussion

Décrit par la première fois en 1907 par James Ramsay Hunt, Le zona otitique est un zona aigu affectant le ganglion géniculé du nerf facial, dû à une manifestation de récurrence (réactivation) du virus varicelle-zona. La distribution mondiale est de 5/100000 habitants/an [1]; le virus du zona a un réservoir strictement humain. Le zona isolé survient sur un terrain sans particularités, pour lequel aucune cause décelable certaine ne peut être affirmée, même si on peut retrouver quelques facteurs favorisants tels que le froid, un traumatisme ou un traitement immunosuppresseur banal, par contre, le zona symptomatique accompagne les affections généralisées graves les hémopathies malignes et le SIDA [2]. Sur le plan histologique, il existe une ballonnisation cellulaire avec inclusions et cellules géantes multi nucléées et une inflammation aiguë du nerf sensitif et du ganglion correspondant avec hémorragies, destructions neuronales et démyélinisation du nerf sensitif. Ces différentes lésions sont responsables d´un arrêt de la conduction nerveuse à un des trois stades de paralysie: neurapraxie axonotmésis neurotmésis.

Le zona auriculaire complet ou syndrome de SICARD, sans être le plus fréquent, c´est le tableau le plus typique et le plus complet de la maladie. Il atteint tous les éléments du paquet nerveux acoustico-facial [2]. Les signes fonctionnels sont marqués par un syndrome infectieux général discret et un syndrome sensitif (otalgie, anesthésie) localisé au niveau de la zone de Ramsay- Hunt, le syndrome éruptif apparait 2 à 4 jours après donnant lieu à des éruptions cutanées caractéristique du zona associées à une réaction ganglionnaire inflammatoire et une hypo ou anesthésie dans la zone de Ramsay Hunt, et une dysgueusie des 2/3 antérieurs de l´hémilangue. Les signes otologiques sont marqués par une surdité unilatérale accompagnée d´acouphènes et/ou d´un syndrome vertigineux rotatoire, et surtout l´installation secondaire d´une paralysie faciale périphérique au 5-6^e^ jour de l´éruption [3]. Cette paralysie faciale (PF) est grave, d´évolution plus sévère que celle de la PF à frigorie. Il existe 4 formes cliniques selon la classification de Ramsay Hunt. Toujours d´actualité, il permet de décrire 4 stades cliniques. Les symptômes peuvent être dissociés de façon variable, la paralysie faciale peut rester totalement isolée, ou associée à quelques douleurs mastoïdiennes mimant une paralysie faciale a frigore, donc devant une paralysie faciale douloureuse il faut savoir évoquer l´étiologie zostérienne. L´atteinte auditive, et vestibulaire, peut survenir sans paralysie faciale, avec ou sans syndrome éruptif, posant le diagnostic différentiel de surdité brusque [4]. Le Diagnostic clinique est souvent facile devant un tableau clinique classique, parfois difficile devant une présentation polymorphe et dissociée, le diagnostic virologique et sérologique prend tout son intérêt devant les formes graves et atypiques [5], le diagnostic virologique et sérologique mis en évidence généralement du virus ou antigènes viraux par des techniques de déviation du complément, immunofluorescence et ELISA [1]. Pour les explorations électrophysiologiques; l´électromyographie renseigne sur le déficit global du nerf, les fibres nerveuses en état de bloc réversible et les fibres en état de dégénérescence, le réflexe stapédien explore les voies aboutissant aux connexions existant entre les structures centrales des noyaux du facial et du nerf auditif [1].

Pour le bilan auditif, l´audiométrie tonale liminaire montre rarement une surdité de transmission, le plus souvent, surdité de perception prédominant sur les fréquences aiguës, la cophose est exceptionnelle; les PEA montrent que la surdité endocochléaire est plus fréquemment retrouvée par rapport aux atteintes rétrocochléaires [1]. Pour le bilan vestibulaire, les épreuves caloriques et rotatoires ne peuvent être faites que si les symptômes spontanés ont disparus, si le patient n´est plus sédaté et ne reçois pas d´antivertigineux, et si le méat auditif est normal. L´atteinte vestibulaire se manifeste par une hypovalence, une hyporéflexie voire une aréflexie [1] qui peut se compenser régresser progressivement. L´imagerie est axée sur l’Imagerie par résonance magnétique (IRM) est un élément topographique déterminant dans la décision opératoire et de son siège, elle permet l´étude des segments intra-axiaux et cisternal du nerf facial dans la fosse postérieure, la tomodensitométrie (TDM) est intéressante pour explorer le canal osseux du facial et la mastoïde [1]. Le zona otitique pose un problème de diagnostic différentiel devant l´éruption impétigo dont l´extension est plus rapide, plus étendue, avec ulcérations suintantes, l´herpès dont les vésicules ne reposent pas sur une base érythémateuse et l´otite externe diffuse simple ou associée à une otite moyenne [1]. Le traitement a pour but de lutter contre le syndrome inflammatoire, la douleur et la paralysie faciale et les troubles cochléo-vestibulaires et d´éviter la diffusion du zona et l´installation de séquelles, [6]. Tous les antiviraux actifs sur le VZV sont des analogues nucléosidiques, agissant par inhibition de l´ADN-polymérase virale qui assure la réplication du virus, ces molécules sont toutes virostatiques et n´agissent que sur les virus en phase réplicative; les 3 molécules disponibles sont l´aciclovir oral à 800 mg, le valaciclovir encore égale au prodrogue de l´aciclovir et famciclovir [6]. L´aciclovir IV est recommandé à la dose de 10 mg/kg chez l´adulte et 500 mg/m^2^ chez l´enfant toutes les 8 heures, pendant une durée minimale de 7 à 10 jours, avec un relais par un traitement per os de 7 jours. La corticothérapie est controversée, la dose est généralement de 1 mg/kg/j en cure courte de 10 J, avec action anti-inflammatoire puissante, bien tolérée, mais une maladie virale en poussée peut être un frein à cette option thérapeutique [6]. Les antalgiques, souvent antalgiques de palier 2, voire 3. Les algies post zostériennes nécessitent l´utilisation d´autres traitements (amitriptyline à 75 mg/j chez l´adulte, carbamazépine (400 à 1 200 mg/j) si paroxysmes hyperalgiques [6]. Les soins locaux sont indispensables pendant la phase aiguë, hygiène cutanée de la zone éruptive par lavage quotidien ou biquotidien. L´instillation de solutions auriculaires antibio-cortisoniques est recommandée en cas d´otite externe et des mesures de protection cornéenne (occlusion, humidification et surveillance). Un examen ophtalmologique s´impose à la moindre sensation d´irritation cornéenne. La rééducation de la paralysie faciale est une urgence, idéalement adressée pour rééducation dans les 15 jours de sa survenue. Elle vise à lutter contre l´hypertonie et les syncinésies. L´aspect psychologique est fondamental car la paralysie faciale est une souffrance [1].

Les attitudes divergent en ce qui concerne la décompression chirurgicale du nerf facial, elle doit être réalisée en regard de la zone de souffrance objectivée par l´imagerie, afin de diminuer le phénomène de garrottage des neurones oedématiées par l´atteinte virale [1]. Les lésions neuronales deviennent irrémédiables après un délai approximatif de 1 mois et la chirurgie est d´autant plus efficace qu´elle est pratiquée avant ce terme. Le traitement doit être entrepris précocement, et inclure dans tous les cas les moyens locaux. Les lésions cutanées cicatrisent en 15 jours alors que la paralysie faciale et les atteintes sensorielles persistent encore. La paralysie faciale zostérienne est réputée de plus mauvais pronostic que la paralysie faciale a frigoré, laissant volontiers des séquelles sous forme de contractures, d´hémispasmes, syncinésies et de syndrome de larmes de crocodile. La surdité est en général de bon pronostic, ne persistant que dans 5% des cas. Il n´existe pas de corrélation entre la sévérité de la paralysie faciale et celle de la surdité. L´atteinte vestibulaire associée aux autres symptômes est élément de mauvais pronostic évolutif pour la paralysie faciale et pour la surdité. De façon générale, les chances de récupération sont meilleures si le traitement est débuté précocement, avant les 72 heures [1], elle peut atteindre 70% de récupération totale. Après 72 heures, seuls 50% des patients récupèrent. Les sujets jeunes semblent avoir une meilleure évolution.

## Conclusion

Le zona otitique est une affection de l'oreille causée par le virus de la varicelle, il survient après réactivation de virus latent au sein du ganglion géniculé, suite à une baisse de l'immunité cellulaire, il représente 4,5% des paralysies faciales périphériques; Le diagnostic repose sur la clinique, dans sa forme typique, il se présente par une éruption cutanée vésiculaire dans la région de Ramsay-Hunt, associé à une paralysie faciale périphérique; on parle alors de syndrome de Ramsay-Hunt. Le traitement repose sur l'association de corticoïde et d'antiviraux associé à la rééducation fonctionnelle précoce; La décompression chirurgicale du nerf facial peut être proposée en cas d'atteinte sévère ayant de mauvais facteurs pronostiques, Une récupération complète surviendrait dans 20 % des cas en l'absence de traitement. Les complications liées à la paralysie faciale sont représentées principalement les syncinésies.
